# Patient‐Specific Coculture of *S. aureus* and *P. aeruginosa* Enhances Epithelial Barrier Disruption and Virulence in CRS

**DOI:** 10.1002/alr.70036

**Published:** 2025-09-30

**Authors:** Xiaohan Sun MMed, Mahnaz Ramezanpour, Jordan Hall, Emma Barry, Alkis J. Psaltis, Peter‐John Wormald, Sarah Vreugde

**Affiliations:** ^1^ Department of Surgery‐Otolaryngology Head and Neck Surgery, Central Adelaide Local Health Network, Woodville South Australia Australia; ^2^ Adelaide Medical School Faculty of Health and Medical Sciences The University of Adelaide Adelaide South Australia Australia

**Keywords:** biofilms, chronic rhinosinusitis, epithelial barrier integrity, polymicrobial infections, *Pseudomonas aeruginosa*, *Staphylococcus aureus*

## Abstract

**Background:**

Chronic rhinosinusitis (CRS) is a chronic inflammatory disease that is associated with polymicrobial infections, often involving *S. aureus* and *P. aeruginosa*. It is unclear whether the polymicrobial context plays a role in exacerbating epithelial damage, inflammation, and resistance to therapy.

**Methods:**

*S. aureus* and *P. aeruginosa* (*n* = 3 each) biofilms were established in a Transwell system, followed by the extraction of *P. aeruginosa* conditioned media and application to an air–liquid interface (ALI) model of human nasal epithelial cells (HNECs). Transepithelial electrical resistance (TEER) and FITC dextran paracellular permeability tests evaluated the epithelial integrity. Colony‐forming unit (CFU) counting, protease activity assay, and pyocyanin and pyoverdine quantification were used to test the proliferation and production of virulence factors of the bacteria.

**Results:**

Cocultures of *P. aeruginosa* and *S. aureus* isolated from the same patient reduced HNEC TEER values, had an earlier onset of HNEC barrier disruption, and increased paracellular permeability compared to monocultures of *P. aeruginosa*. *P. aeruginosa* proliferation was enhanced, and protease activity increased significantly. The production of pyoverdine increased significantly in the same patient cocultures, while the pyocyanin levels remained unchanged.

**Conclusions:**

These results indicate a role of within‐host evolution in shaping *P. aeruginosa*‐mediated virulence in the context of polymicrobial biofilms. This supports the need to develop strategies directed at disrupting interspecies synergies that culminate in the formation of polymicrobial biofilms associated with CRS for the purpose of improving disease management and therapeutic efficacy.

## 1 Introduction

Polymicrobial infections represent a key feature of numerous chronic inflammatory disorders, most notably chronic rhinosinusitis (CRS), and can involve multiple bacterial species, whose complex and dynamic relationships impact disease progression, severity, and therapeutic efficacy [1]. *S. aureus* and *P. aeruginosa* are among the more frequently recovered bacterial species in CRS patients, particularly in severe therapy‐refractory CRS [2]. Both species have received considerable attention for their capacity for survival in the sinonasal environment and for potential interactions in polymicrobial biofilms. Having both pathogens present in a shared biofilm brings important questions regarding disease chronicity, persistence, and therapeutic resistance [1].

CRS is a long‐standing inflammatory disease of the nasal and sinus mucosa, seen in 10% of the population worldwide [3]. Although the etiopathogenesis is not precisely understood, microbial infection is considered a key player in inducing and perpetuating inflammation [4]. Polymicrobial biofilms have been seen to prevail in a widespread manner, extending over the sinonasal mucosa in CRS subjects, and most predominantly in subjects having a high disease burden. These biofilms serve as a protective niche for bacteria, allowing them to evade host immune responses and resist therapeutic actions, making effective treatment enormously challenging [5, 6, 7]. Existing clinical and mechanistic studies suggest that the coexistence of *S. aureus* and *P. aeruginosa* in the same multispecies biofilm may exacerbate the clinical phenotype of CRS and reduce the efficacy of conventional treatment. *S. aureus* colonization is associated with postoperative recurrence and poor prognosis [8], while the exoprotein activity of *P. aeruginosa* is associated with disease exacerbation and epithelial barrier disruption [9]. Despite limited direct evidence, these two bacteria are often detected simultaneously in refractory CRS characterized by persistent biofilms [10].

A unique feature of polymicrobial biofilms is interspecies interactions. Physical nearness, metabolic reciprocity, and chemical signaling networks, including quorum sensing (QS), mediate these interactions [11]. QS functions as a microbial communication network that controls bacterial gene expression through population density while facilitating collective behavior processes, including virulence factor expression, biofilm formation, and emergence of antibiotic resistance [12]. *P. aeruginosa* secretes multiple QS molecules, which include both pyoverdine and pyocyanin [13]. Microorganisms use siderophore molecules such as pyoverdine to capture iron in the environment, unlocking bacterial growth and virulence. In addition to inducing tissue damage through oxidative stress processes, pyoverdine at high concentrations can compromise epithelial barrier function [14]. High concentrations of pyoverdine are often found in homologous cocultures, indicating that interspecies cooperation maximizes the availability of iron for acquisition. This is important, especially in the context of infection, where the host induces a “nutritional immunity” response aimed at reducing the amount of iron available for invading microorganisms [15]. The competition for iron is a universally acknowledged impetus for microbial interactions, shaping the composition and behavior of polymicrobial communities [16]. The virulence factor pyocyanin acts as a multifaceted compound that executes direct cytotoxicity. Production of reactive oxygen species (ROS) by pyocyanin triggers oxidative damage in host cells with inhibition of their immune function [17]. Study results reveal widespread complex interactions and virulence regulation of *P. aeruginosa* and *S. aureus* during homologous culture in polymicrobial biofilms [18]. Polymicrobial infection studies demand careful observation of microbial behavior specific to the host since disease progression in a patient hinges on interspecies interaction in a polymicrobial environment.

Protease activity, involved in invasion and evasion of immunity, is a significant mechanism modulated by interspecies interactions [19]. This activity destroys host extracellular matrix structures, compromising tissue integrity and promoting bacterial dissemination [20]. Protease activity disrupts barrier function in epithelial layers, as evidenced by reduced transepithelial electrical resistance (TEER) and augmented paracellular permeability in model experiments [19].

Despite these important findings, the mechanism of *S. aureus* and *P. aeruginosa* interaction in the context of CRS is relatively unknown. Defining such mechanisms is imperative for developing therapies that target the specific challenge of polymicrobial infection. Inhibiting the QS processes while targeting individual virulence factors that maintain interspecies relations has the potential to degrade biofilm infrastructure, thus improving the effectiveness of standard antimicrobial treatments [21].

This study investigates the interactions between *S. aureus* and *P. aeruginosa* obtained from CRS patients, focusing on the impact of *S. aureus* biofilm on *P. aeruginosa* biofilm formation, epithelial barrier integrity, and virulence factor production. By comparing homologous and heterologous cocultures, this research aims to uncover the patient‐specific dynamics that shape these interactions. Insights from this research expand our understanding of polymicrobial infections in CRS while providing directions for creating improved therapeutic approaches for enhanced treatment results and improved patient well‐being.

## 2 Materials and Methods

### 2.1 Sourcing of Bacterial Strains and Ethics Statement

Ethics approval for the collection, storage, and use of clinical isolates and primary human nasal epithelial cells (HNECs) was granted by The Central Adelaide Local Health Network (CALHN) Human Research Ethics Committee (HREC) (CALHN Ref. 13604) and the Calvary Hospital HREC [Ref. 19‐CHREC‐E003], both in Adelaide, South Australia, and all patients had signed written informed consent. Patients with CRS met the diagnostic criteria as outlined in the latest position papers from the American Academy of Otolaryngology and Head and Neck Surgery and the European Position Statement (EPOS) on CRS [3]. *S. aureus* and *P. aeruginosa* had been isolated, identified by Matrix‐Assisted Laser Desorption Ionization‐Time of Flight Mass Spectrometry (MALDI‐TOF MS, Bruker MBT, The University of Adelaide, Adelaide, Australia), and stored at −80°C in tryptone soy broth (TSB, Oxoid, Basingstoke, UK) plus 20% (v/v) sterile glycerol until further use.

Patient demographics and clinical characteristics of the CRS cohort are summarized in Table 1. All isolates were obtained from three adult patients diagnosed with CRS, with variations in comorbidities such as asthma and gastroesophageal reflux disease (GORD). CRS phenotypes (with vs. without nasal polyps) and disease severity scores (Adelaide Disease Severity Score [ADSS] [22], ranging from 0 to 32) were recorded where available. In all cases, P. aeruginosa and S. aureus were co‐isolated from the same clinical swab at the time of sampling. This table provides a concise overview of key variables relevant to inter‐patient comparability.

**TABLE 1:** Clinical and demographic characteristics of patients from whom CRS isolates were obtained.

Patient ID	Age (years)	Sex	CRS phenotype	Smoking history	Allergies	Asthma	GORD	DM	ADSS score
6027	82	F	CRSsNP	Nonsmoker	None	Yes	Yes	No	25
539	60	F	CRSwNP	Nonsmoker	Yes	Yes	Yes	Yes	14.5
1415	53	M	CRSwNP	Unknown	None	No	Yes	Yes	Not recorded

Abbreviations: ADSS, Adelaide Disease Severity Score; CRS, chronic rhinosinusitis; CRSsNP, CRS without nasal polyps; CRSwNP, CRS with nasal polyps; DM, diabetes mellitus; F, female; GORD, gastroesophageal reflux disease; M, male.

### 2.2 Collection and Cultivation of Primary HNECs

Primary HNECs were harvested from the middle turbinates of patients with clinical evidence of CRS under endoscopic guidance. Samples were collected using gentle brushing techniques and then suspended in Nasal Epithelial Growth Media (STEMCELL Technologies Australia Pty. Ltd., Tullamarine, VIC, Australia). To deplete monocytes, the extracted cells were cultured in dishes coated with anti‐CD68 antibodies (Dako, Glostrup, Denmark). HNECs were expanded under standard cell culture conditions at 37°C in a humidified atmosphere with 5% CO_2,_ using collagen‐coated flasks (Thermo Scientific, Waltham, Massachusetts, USA) in PneumaCult‐EX Plus complete medium (Stemcell Technologies, 05001). The cells were utilized at Passage 1 for subsequent experiments [23].

### 2.3 Air–Liquid Interface Culture

HNECs were maintained in air–liquid interface (ALI) culture using PneumaCult‐ALI medium (Stemcell Technologies, 05001, Cambridge, UK) according to the recommended protocol [23]. HNECs were grown in collagen‐coated flasks until they reached approximately 80% confluence, after which they were harvested and seeded onto collagen‐coated 6.5‐mm permeable Transwells (BD Biosciences, San Jose, California, USA) at a density of 7 × 10^4^ cells per well. The cell cultures were maintained in PneumaCult‐EX Plus complete medium for 3–4 days in a cell incubator set at 37°C with 5% CO_2_. During the differentiation step, the apical medium was removed, and the basal medium was replaced with 500 µL of PneumaCult‐ALI medium. The cultures were then fed every other day by adding PneumaCult‐ALI medium to the basal chamber. HNECs were maintained at the ALI (HNEC–ALI) for a minimum of 14 days to facilitate the development of tight junctions.

### 2.4 Mixed‐Species Bacterial Biofilm Coculture and Measurement of Conditioned Media Concentration

*Staphylococcus aureus* (C317, C318, C324) and *Pseudomonas aeruginosa* (C440, C442, C443) clinical isolates obtained from CRS patients were grown overnight on tryptic soy agar (TSA, Oxoid, Basingstoke, UK) plates. Colonies were transferred into a sterile glass tube containing 0.9% sodium chloride and adjusted to a 1.0 ± 0.1 McFarland turbidity standard (approximately 3 × 10^8^ colony‐forming units [CFU]/mL). The bacterial suspension was diluted 1:15 in nutrient broth. Then, 500 µL of the final *P. aeruginosa* suspension was added to the basal chambers of Transwell 24‐well plates (Corning, New York, USA). To the apical chambers, 100 µL of the same *P. aeruginosa* strain was added to serve as the control group, while 100 µL suspensions containing *S. aureus* from either the same patient or different patients constituted the experimental group. The plates were then incubated at 37°C for 48 h on a gyratory mixer (3D Gyratory Mixer; Ratek Instruments, Boronia, Australia) at 70 rpm. Supernatants from the basal chambers were collected and centrifuged at 1500 × *g* for 10 min at 4°C. Conditioned media protein concentrations were measured using the Nano‐orange protein quantitation kit (Molecular Probes, Eugene, Oregon, USA) according to the manufacturer's instructions. The fluorescence intensity was measured with excitation at 485 nm and emission at 590 nm using the FLUOstar Optima microplate reader (BMG Lab Tech).

### 2.5 Determination of CFU of Cocultured Bacteria

200 µL liquid bacterial culture from the basal chamber of Transwell plates was transferred to wells in a flat‐bottom 96‐well microtiter plate, and 10‐fold serial dilutions were prepared in PBS. 20 µL drops of each dilution were spotted in triplicate onto one quarter of a *Pseudomonas* cetrimide agar plate (Oxoid, UK), before incubating overnight at 37°C. The following day, colonies were enumerated, and CFU/mL was calculated by multiplying the average count by the dilution factor and a volume factor of 50. All samples were subjected to triplicate measurements to verify the reliability.

### 2.6 Measurement of TEER

TEER was measured using an EVOM2 epithelial Voltohmmeter (World Precision Instruments, Sarasota, FL). HNEC‐ALI cultures were used only when TEER values at baseline exhibited a resistance greater than 400 Ω/cm^2^. Subsequently, 100 µL of PneumaCult‐ALI medium (negative control), 10% Triton X‐100 in PneumaCult‐EX Plus complete medium (positive control), or bacterial conditioned media, adjusted to 5 µg/mL with PneumaCult‐EX Plus complete medium to a total volume of 100 µL, were added to the apical chamber of the ALI cultures. TEER measurements were taken at various time points: 0, 0.5, 1, 2, 3, 4, 5, 6, and 24 h after treatment application. The cultures were maintained at 37°C during the measurement period on a heating platform. To ensure reproducibility, experiments were repeated four times.

### 2.7 FITC‐Dextran Permeability Assay

After measuring the TEER value at the 24‐h incubation time point, the paracellular permeability of HNEC‐ALI cultures was evaluated by aspirating the medium from the apical chambers and rinsing them once with PBS. Following this, the PBS was removed, and 10 µL of 4‐kDa fluorescein isothiocyanate (FITC)‐dextran (101617294, Sigma, Saint Louis, USA) at a concentration of 3 mg/mL was added, along with 90 µL of PneumaCult‐EX Plus complete medium, to the HNEC‐ALI cultures. The plate was then incubated at 37°C for 2 h, protected from light. Subsequently, 40 µL samples were taken from the basal chamber and transferred to a black 96‐well plate (167008, Corning‐Costar Corp., Cambridge, UK), with each sample, including negative and positive controls, tested in duplicate. Finally, fluorescence was measured using a FLUOstar Optima 96‐well fluorescence microplate reader (BMG Labtech, Ortenberg, Germany) set to an excitation wavelength of 485 nm and an emission wavelength of 520 nm. Experiments were repeated four times.

### 2.8 Evaluation of Pyoverdine and Pyocyanin Production

To evaluate pyoverdine production, 250 µL of supernatant from cocultures of *S. aureus* and *P. aeruginosa*, as well as from *P. aeruginosa* monocultures, was added to Luria‐Bertani (LB) agar overnight. A loopful of bacteria was then transferred into LB liquid medium and incubated for an additional 24 h. Following this, 100 µL of the culture was transferred to a black 96‐well microplate, with each sample tested in duplicate. Pyoverdine fluorescence was measured using a CLARIOstar Plus microplate reader (BMG Labtech, Offenburg, Germany) at an excitation wavelength of 405 nm and an emission wavelength of 460 nm.

To measure pyocyanin, 250 µL of supernatant from cocultures of *S. aureus* and *P. aeruginosa*, as well as from *P. aeruginosa* monocultures, was cultured on nutrient agar plates overnight. Following incubation, a loopful of bacteria was inoculated into nutrient broth and incubated for 24 h to promote metabolite production. The resulting bacterial culture was then centrifuged at 4255 × *g* for 15 min at 4°C. The supernatant was collected. An equal volume of chloroform (4.5 mL) was added to 4.5 mL of the supernatant (1:1, v/v), and the mixture was vortexed for 30 s. Subsequently, the mixture was centrifuged again at 4255 × *g* at 4°C for 10 min, and the chloroform layer was transferred to a new tube. Next, 1.5 mL of 0.2 mol/L HCl was added to the 4.5 mL chloroform layer (resulting in a chloroform:HCl ratio of 3:1, v/v), mixed thoroughly, and centrifuged for an additional 10 min. The HCl layer was then collected, and 100 µL of this layer was transferred to a black 96‐well microplate and tested in duplicate. Absorbance was measured at OD 520 nm using a CLARIOstar Plus microplate reader (BMG Labtech). All experiments were performed in triplicate.

### 2.9 Measurement of Protease Activity in Cocultured Bacteria

To evaluate bacterial protease activity, 30 µL of supernatant from a coculture of *P. aeruginosa* and *S. aureus* or supernatant of monocultures of *P. aeruginosa* was spotted in a single spot onto a skim milk agar plate (Oxoid, Basingstoke, UK) and incubated at 37°C for 24 h. After incubation, a ruler was used to measure the diameter of the resulting bacterial colony and its surrounding clear zone (halo), with results recorded in centimeters (cm). The clear zone around the colony, indicative of casein hydrolysis, signified positive protease activity. The experiment was performed in quadruplicate.

### 2.10 Statistical Analysis

Statistical analyses were conducted using GraphPad Prism (version 10.0; GraphPad Software, San Diego, California, USA). One‐way ANOVA, two‐way ANOVA, and Tukey's test were applied to evaluate statistical differences. A *p* value of < 0.05 was considered to indicate statistical significance.

## 3 Results

### 3.1 *S. aureus* Biofilm Augments *P. aeruginosa* Biofilm‐Mediated Mucosal Barrier Dysfunction When Isolates Are Isolated From the Same Patient

We first wanted to evaluate whether indirect interactions between biofilms of *S. aureus* and *P. aeruginosa* isolated from the same or from different patients affected *P. aeruginosa*‐induced effects on the mucosal barrier compared to *P. aeruginosa* monoculture biofilms. Three pairs of bacterial strains, isolated from the sinonasal cavities of three CRS patients, were used in these experiments. *S. aureus* (or *P. aeruginosa* control) and *P. aeruginosa* biofilms were established in the upper and lower chambers of Transwell plates, respectively, followed by extraction of conditioned media from the lower chamber. Conditioned media or controls were applied to HNEC‐ALI cultures, followed by measuring TEER at multiple time points over 24 h.

**FIGURE 1:**
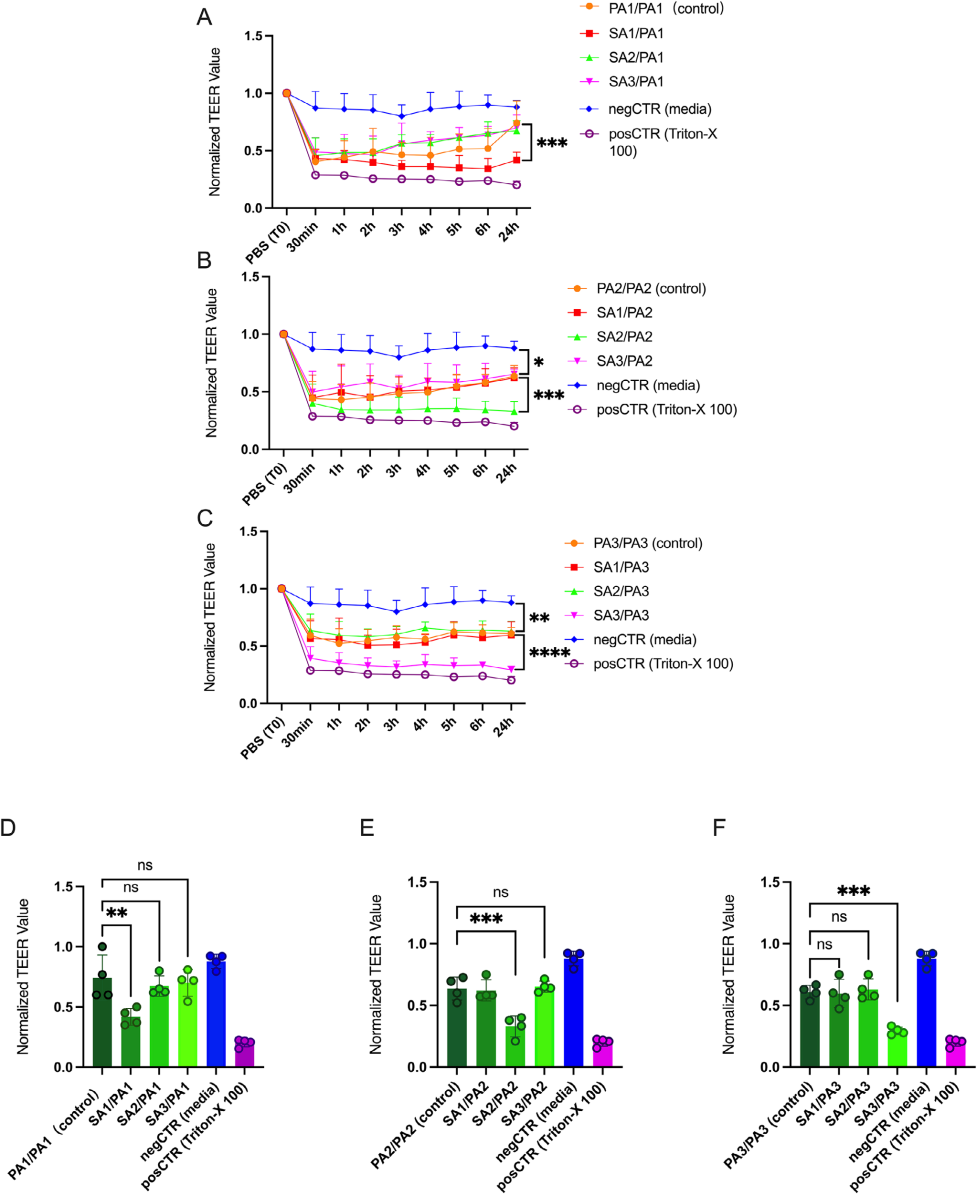
Normalized TEER Values in cocultures and monocultures. (A–C) Time‐course analysis of normalized TEER values in monocultures of *P. aeruginosa* and cocultures of *S. aureus* from same or different patients with *P. aeruginosa* for patient 1 (A), 2 (B), and 3 (C). (D–F) Endpoint analysis of normalized TEER values at 24 h in monocultures of *P. aeruginosa* and cocultures of *S. aureus* from the same or different patients and *P. aeruginosa* for patient 1 (D), 2 (E), and 3 (C). PA, *P. aeruginosa*; PA–SA, *P. aeruginosa* is cultured in the lower chamber while *S. aureus* is cultured in the upper chamber; SA, *S. aureus*. Statistical significance was determined using two‐way ANOVA and one‐way ANOVA followed by Tukey's multiple comparisons test; ns (not significant), *p < 0.05, **p < 0.01, ***p < 0.001, ****p < 0.0001.

We first established cocultures with *P. aeruginosa* and *S. aureus* isolated from the same patients. For all three patients, equal concentrations of *P. aeruginosa* biofilm conditioned media from cocultures with *S. aureus* biofilms resulted in significantly lower TEER values compared to *P. aeruginosa* monocultures at the 24‐h timepoint. A two‐way ANOVA was performed across all time points to assess statistical significance, although significance markers (asterisks) are only shown for the 24‐h timepoint in Figure 1A–C for clarity. Detailed time‐course analysis revealed that all samples exhibited a reduction at the 30‐min timepoint; however, the onset of a statistically significant decrease in TEER varied among individual samples. The onset and magnitude of TEER reduction varied among individual patients, as detailed in Table S1.

We then wanted to evaluate whether similar effects would be seen when *P. aeruginosa* and *S. aureus* had been isolated from different patients. *P. aeruginosa* biofilms were established for all three patients in the presence or absence of *S. aureus* from the other two patients. *P. aeruginosa* biofilm conditioned media from cocultures with *S. aureus* biofilms showed no significant difference in TEER compared to *P. aeruginosa* monoculture at any time point for any of the patients when both species were isolated from different patients (*p* > 0.05).

These findings suggest that *P. aeruginosa*‐dependent epithelial barrier damage is more pronounced when cocultured with *S. aureus* isolated from the same patient compared to cocultures with *S. aureus* from a different patient. This trend is consistently observed across both the time‐course data (Figure 1A–C) and the 24‐h endpoint comparison (Figure 1D–F), although variability in the onset and magnitude of disruption is evident.

**FIGURE 2:**
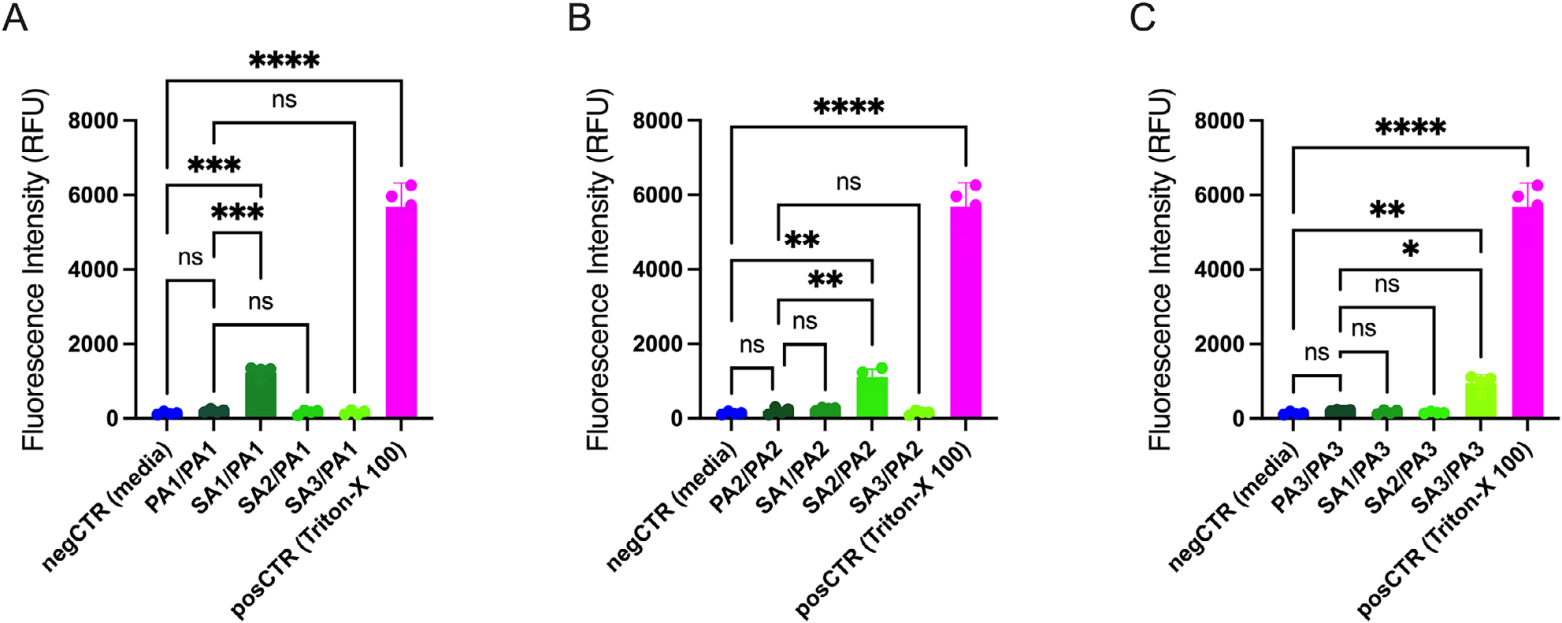
Paracellular Permeability of HNEC‐ALI Cultures Measured by FITC‐dextran Permeability Assay. Fluorescence intensity in the basal chamber for HNEC‐ALI cultures exposed to *P. aeruginosa* monocultures, cocultures of *S. aureus* and *P. aeruginosa* from the same patient, or cocultures involving isolates from different patients for Patient 1 (A), Patient 2 (B) and Patient 3 (C). Fluorescence was measured after a 2‐h incubation with FITC‐dextran. PA, *P. aeruginosa*; PA–SA, *P. aeruginosa* is cultured in the lower chamber while *S. aureus* is cultured in the upper chamber, SA, *S. aureus*. Statistical significance was determined using one‐way ANOVA and Tukey's test. ns (not significant), *p < 0.05, **p < 0.01, ***p < 0.001, ****p < 0.0001.

The paracellular permeability of HNECs cultured at the ALI was then evaluated through FITC‐dextran permeability assays conducted subsequent to the measurement of TEER values at the 24‐h incubation time point. Cocultures of *S. aureus* and *P. aeruginosa* strains isolated from the same patient resulted in a 4.3–6.1‐fold higher FITC‐dextran permeability compared to *P. aeruginosa* single‐species cultures or cocultures involving strains from different patients (*p* < 0.05) for all three patients (*p* < 0.05). In contrast, the fluorescence intensity after application of exoproteins from single‐species *P. aeruginosa* cultures or from *P. aeruginosa* cocultured with *S. aureus* from different patients was not different from the negative control for any of the patients tested (*p* > 0.05). Results are shown in Figure 2 and Table S2.

These results indicate that cocultures of *S. aureus* and *P. aeruginosa* biofilms from the same patient enhance the paracellular permeability.

### 3.2 Indirect Interactions Between *S. aureus* and *P. aeruginosa* Biofilms Isolated From the Same Patient Enhance *P. aeruginosa* CFU

**FIGURE 3:**
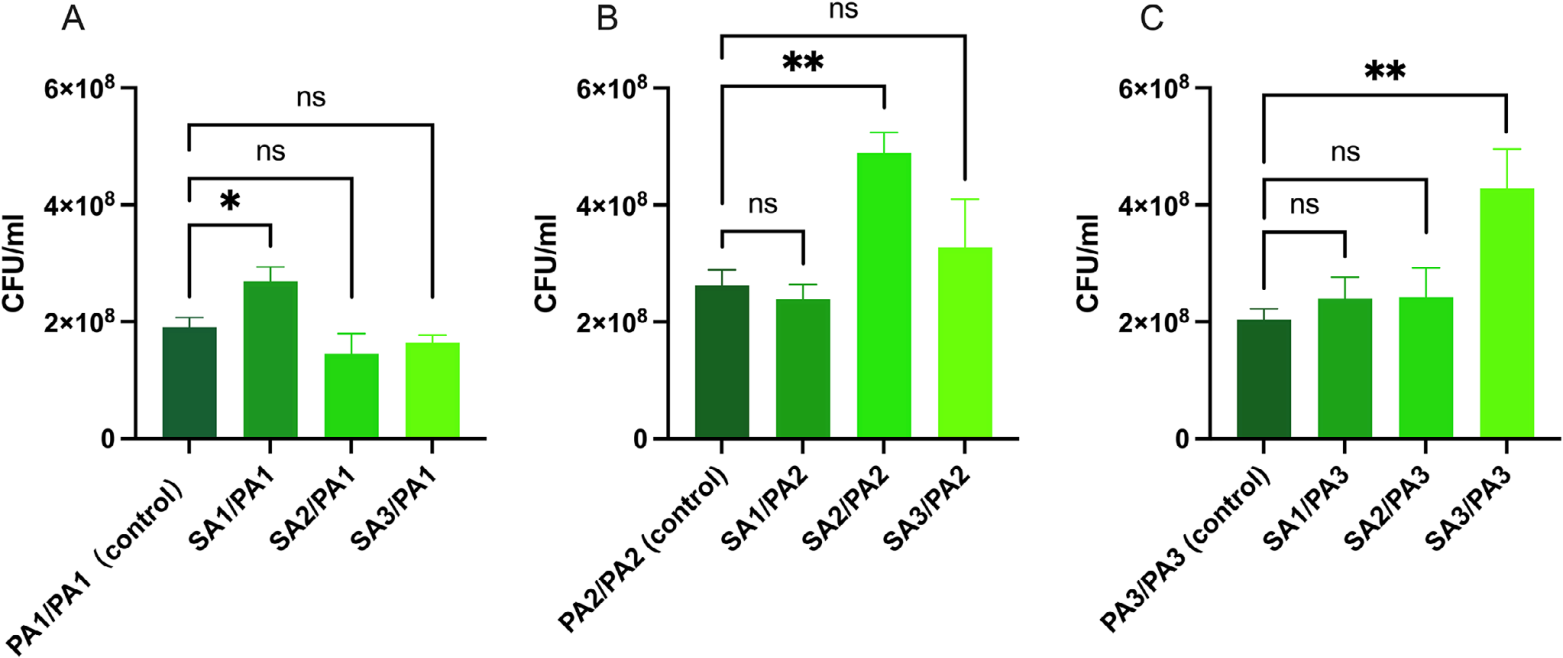
Colony‐forming units (CFU) in cocultures of *S. aureus* and *P. aeruginosa* and in *P. aeruginosa* monocultures. (A) CFU counts for Patient 1; (B) CFU counts for Patient 2; (C) CFU counts for Patient 3. Statistical significance was determined using one‐way ANOVA followed by Tukey's multiple comparisons test. ns: not significant, *p < 0.05, **p < 0.01.

To assess bacterial viability and proliferation in cocultures, CFU were quantified for bacterial cultures from the lower chambers of Transwell systems. *P. aeruginosa* biofilm in indirect contact with *S. aureus* biofilm isolated from the same patient exhibited significantly higher CFU counts compared to single‐species *P. aeruginosa* cultures or cocultures with *S. aureus* from different patients (*p* < 0.05) for all three patients (Figure 3). CFU counts for all conditions are detailed in Table 3.

These results suggest that *P. aeruginosa* biofilm, when in indirect contact with *S. aureus* biofilm from the same patient, promotes significantly higher bacterial proliferation compared to monocultures.

### 3.3 *P. aeruginosa* Biofilm Pyoverdine Production Is Increased in Cocultures of *S. aureus* and *P. aeruginosa* Isolated From the Same Patient

**FIGURE 4:**
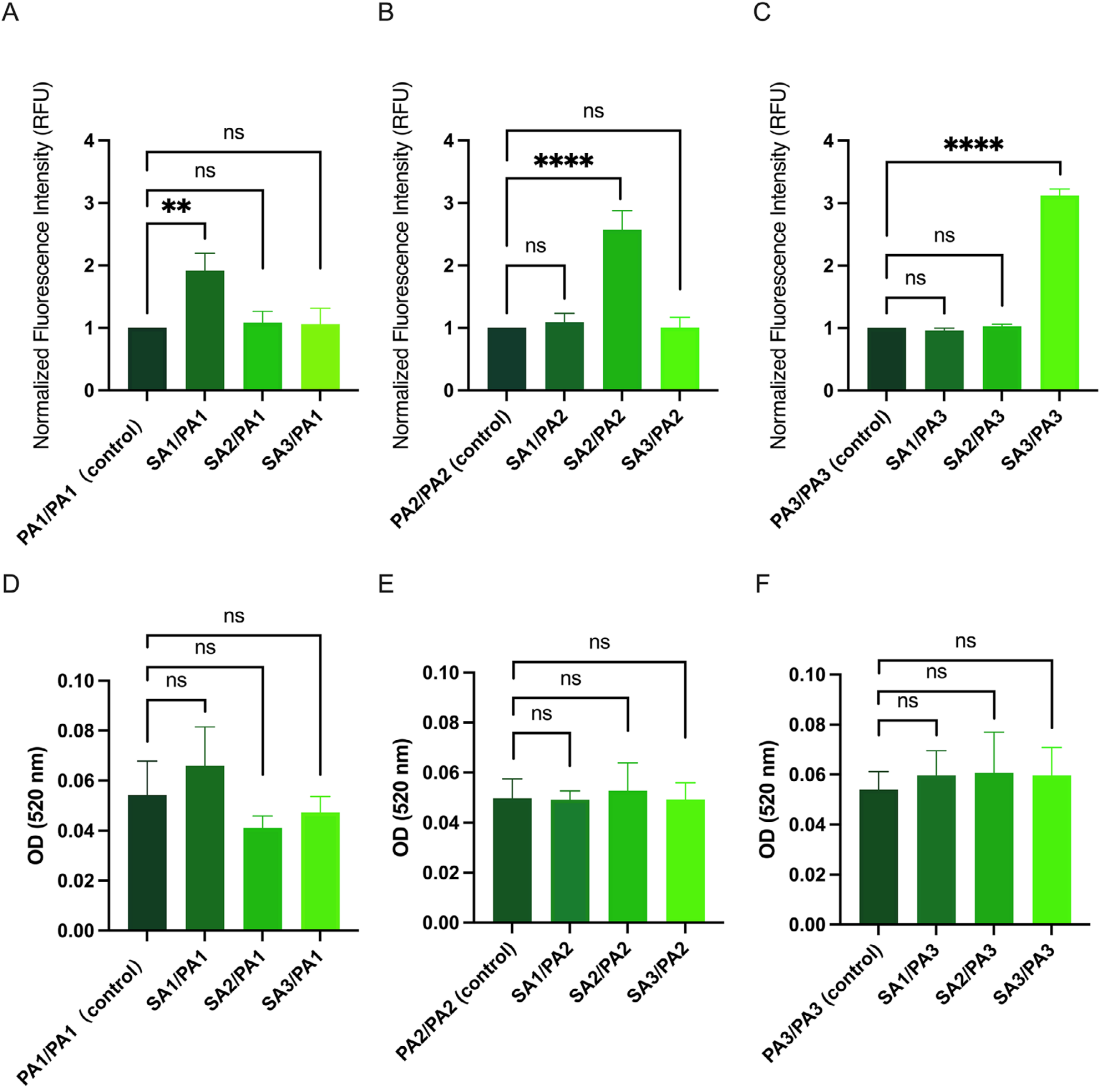
Impact of coculture on pyoverdine and pyocyanin production by *P. aeruginosa*. Pyoverdine production measured by normalized fluorescence intensity (RFU) (A–C) and pyocyanin production measured by absorbance at OD 520 nm (D–F) in cocultures of *S. aureus* and *P. aeruginosa* isolates or the monoculture of the *P. aeruginosa* isolated from patient 1 (A, D), 2 (B, E), and 3 (C, F). PA, *P. aeruginosa*. SA, *S. aureus*, Statistical significance was determined using one‐way ANOVA followed by Tukey's multiple comparisons test: ns (not significant), **p < 0.01, ****p < 0.0001.

To investigate the influence of *S. aureus* biofilm on the *P. aeruginosa* biofilm‐mediated production of pyoverdine and pyocyanin, supernatants from cocultures and single‐species cultures were analyzed using fluorescence‐ and absorbance‐based assays, respectively. Results showed that pyoverdine production was significantly enhanced in cocultures involving isolates from the same patient compared to single‐species cultures (*p* < 0.05) for all three patients (Figure 4A–C). In contrast, cocultures with *S. aureus* from different patients did not increase *P. aeruginosa* pyoverdine production compared to monocultures (*p *> 0.05). Pyocyanin levels remained unaffected by coculture conditions, whether cocultures of *S. aureus* were from the same or from different patients (*p* > 0.05) (Figure 4D–F).

### 3.4 The Effect of Cocultured *S. aureus* and *P. aeruginosa* Isolates on *P. aeruginosa* Protease Activity in CRS Patients

Protease activity in bacterial cultures was evaluated by measuring the diameters of bacterial colonies and their surrounding clear zones (halos) on skim milk agar plates. These halos, representing casein hydrolysis, served as an indicator of protease activity. The study analyzed cocultures of *S. aureus* and *P. aeruginosa* isolates derived from the same and different CRS patients, with single‐species *P. aeruginosa* cultures included as controls for comparison.

**FIGURE 5:**
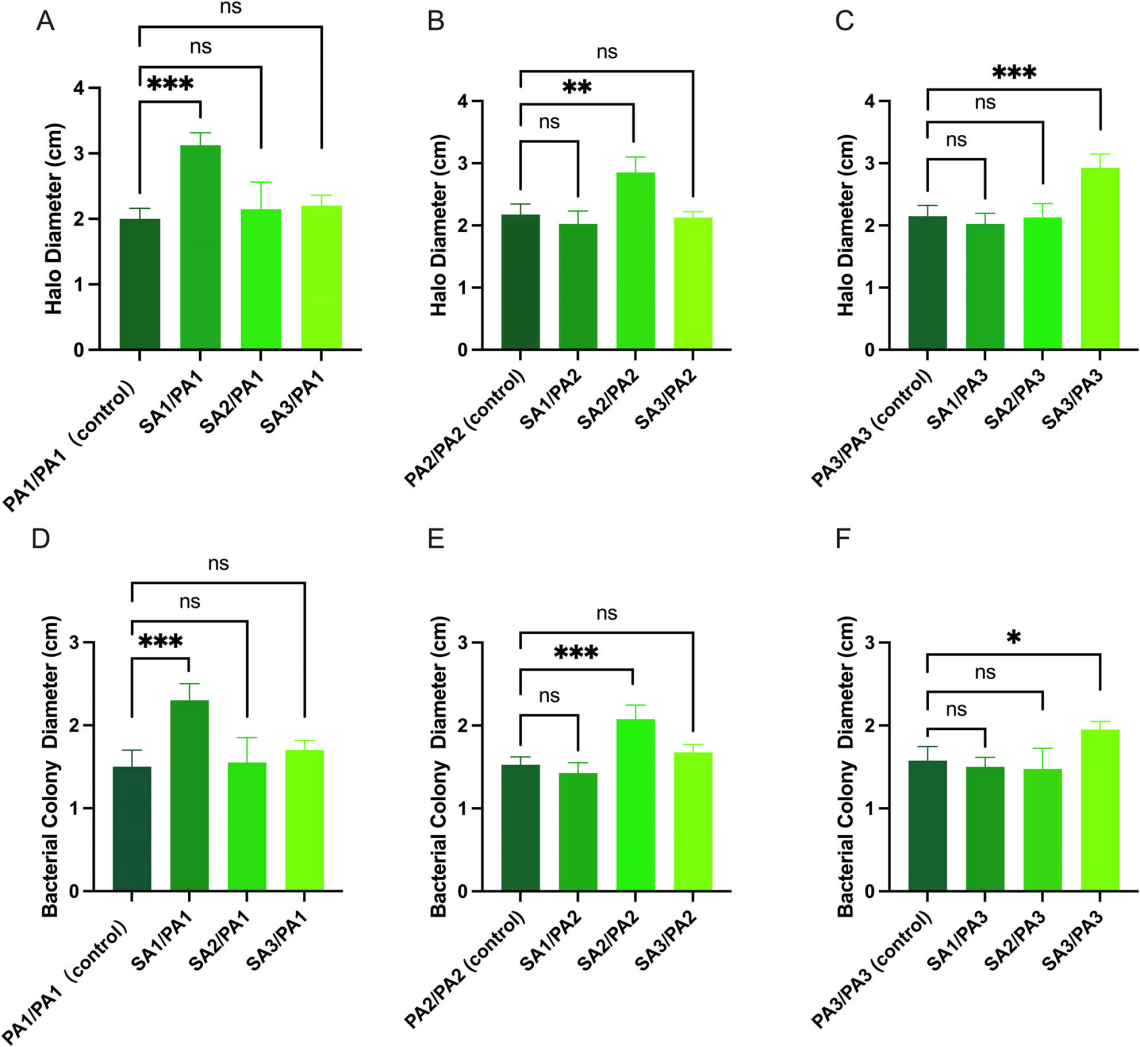
Impact of coculture on protease activity and bacterial growth. (A–C) clear zone diameters from cocultures of *S. aureus* and *P. aeruginosa* or monocultured *P. aeruginosa* from Patients 1–3. (D–F) Colony diameters from the same culture conditions. PA, *P. aeruginosa*; SA, *S. aureus*. Statistical significance was determined using one‐way ANOVA followed by Tukey's multiple comparisons test. ns (not significant), *p < 0.05, **p < 0.01, ***p < 0.001.

For patient 1, *P. aeruginosa* cocultured with *S. aureus* from the same patient produced clear zones with a significantly larger mean diameter compared to those formed in monocultures. In contrast, when *S. aureus* isolates from Patients 2 and 3 were cocultured with *P. aeruginosa* from Patient 1, the resulting clear zone diameters were comparable to those observed in *P. aeruginosa* monocultures, with no significant differences. A similar pattern was observed for Patients 2 and 3, where cocultures involving *S. aureus* and *P. aeruginosa* isolates derived from the same patient generated significantly larger clear zones than *P. aeruginosa* monocultures (*p* < 0.05). However, cocultures involving *P. aeruginosa* and *S. aureus* isolates from different patients did not result in increased clear zone diameters, which remained similar to those of monocultures (Figure 5).

In addition to clear zone measurements, cocultures of *S. aureus* and *P. aeruginosa* isolated from the same patient also led to a significant increase in bacterial colony size, suggesting enhanced growth dynamics. In contrast, mixed‐species cultures involving isolates from different patients yielded colony diameters comparable to those of single‐species cultures, with no statistically significant differences (*p* > 0.05). (Figure 5D–F). Detailed quantitative comparisons of colony and clear zone diameters across all experimental groups are provided in Tables S4 and S5.

## 4 Discussion

In this study we focused on *S. aureus* and *P. aeruginosa* biofilm interactions, showing enhancement of *P. aeruginosa* biofilm‐mediated epithelial barrier impairment, an increased bacterial proliferation, and enhanced virulence factor production when those biofilms were in indirect contact with *S. aureus* biofilms. These findings were consistently seen only when both species were isolated from the same patient, indicating the importance of host adaptation in specific interspecies interactions. The study provides insights into the role of interspecies interactions and how they might promote CRS disease recalcitrance.

The current study shows that homologous bacterial coculture systems involving *S. aureus* and *P. aeruginosa* induce significantly enhanced *P. aeruginosa* biofilm‐mediated defects in the epithelial barrier, evidenced by a reduction in TEER and concurrent increased paracellular permeability. These measures of epithelial structural health serve as vital markers for mucosal barrier function, and their alteration signals major damage to these protective membranes [24, 25]. Estimates of TEER reduction from patient‐derived isolates showed divergence in onset and magnitude of barrier deterioration, where some strains affected the barrier after only 30 min, while others required 24 h of exposure. This variation indicates differences in the production of factors that affect the barrier in either quantity or quality, potentially associated with specific genetic characteristics of the bacterial isolates and how they regulate the dynamics of interaction [26]. Differences such as these could also represent adaptive processes specific to each patient's microbial and host environment, in which long‐term coexistence promotes interspecies cooperation [27]. While both species were each time isolated from the same niche in three CRS patients, it is unknown how long they coexisted in the same patient prior to sampling. Further experiments are required to investigate interspecies interactions in species isolated from the same patient over time to further investigate the role of pathoadaptive processes in enhancing *P. aeruginosa* biofilm‐mediated virulence in the context of polymicrobial infections.

The consequences of such increased epithelial barrier disruption have widespread implications. The epithelial barrier is the first and primary barrier to microbial invasion and immune stimulation. Compromised, it promotes the translocation of bacteria and virulence factors deeper into tissue, inducing inflammation and disease severity [28]. This specificity underlines the necessity of patient‐specific approaches in CRS therapy, in which blocking of specific microbial interaction could counteract epithelial damage and possibly reverse barrier dysfunction [29].

Our study also shows that homologous cocultured *P. aeruginosa* have elevated bacterial population growth, while heterologous cocultures do not, further emphasizing the importance of host adaptation in these cooperative mechanisms. This could be due to resource‐sharing dynamics, where metabolic byproducts from one species serve as substrates for the other [30]. These bacteria share nutrients through metabolism, which could help them survive within the nutrient‐scarce conditions found in sinonasal cavities. This metabolic interaction gives them a greater chance to escape both host defenses and existing antimicrobial treatments [31].

The function of virulence factors in modulating such interactions is a key theme of this work, shedding light on processes in which *P. aeruginosa* optimizes its pathogenicity in coculture environments. Among such factors, the production of pyoverdine, a siderophore important in iron capture, was significantly elevated in homologous cocultures of *P. aeruginosa*. Iron is an important substrate for both pathogen and host cells and is at the epicenter of numerous processes, including DNA synthesis, respiration, and oxidative stress maintenance [32]. The increase in pyoverdine production serves not only as an indicator of heightened virulence in *P. aeruginosa* but may also contribute to enhanced pathogenicity by promoting improved iron acquisition and cooperative adaptation among interacting bacterial strains [33]. Such a strategy could confer a considerable edge for *P. aeruginosa* in CRS, in which the immune system of the host places extreme restrictions on microbial access to iron through processes such as nutritional immunity [34].

The function of pyoverdine is not restricted to iron acquisition. This siderophore can indirectly contribute to bacterial competitiveness through its generation of ROS in the host environment, contributing to heightened oxidative stress and tissue degradation [15]. Through both its role in nutrient retrieval and degradation of host tissue, increased production of pyoverdine in homologous cocultures reveals the pathogenicity of cooperative bacterial behavior.

Conversely, the production of pyocyanin, another characterized virulence factor of *P. aeruginosa*, was not affected by coculturing, indicating selective modulation of virulence pathways. Pyocyanin, a blue‐green phenazine pigment, is a multi‐action virulence factor in bacterial pathogenicity. It is a strong, redox‐active compound that disables host cell activity through ROS production, damaging cellular components such as lipids, proteins, and DNA [35]. Pyocyanin disables host immune function by interfering with processes such as phagocytic function, T‐cell stimulation, and cytokine production, apart from its ROS generation‐dependent activity [17]. Although pyocyanin plays a proven role in infections caused by *P. aeruginosa*, its production in cocultures was not different to monocultures, showing a more stable output and lower susceptibility to interspecies interactions compared to pyoverdine.

This contrast could arise out of variance in the networks controlling the biosynthesis of pyoverdine and pyocyanin. Production of pyoverdine is iron‐regulated via a ferric uptake regulator (Fur) system, which could respond dynamically to interspecies competition in homologous cocultures [36, 37]. In contrast, pyocyanin biosynthesis is modulated by the QS systems, specifically las and rhl [38, 39], whose activity might not be significantly impacted by the presence of *S. aureus* biofilms under experimental conditions of the current study.

Our findings carry major implications that affect clinical practice. Biofilms present protective environments for bacteria that survive both antibiotics and immune actions and therefore make the disorder challenging to cure [40]. *S. aureus* biofilm‐mediated effects on enhancing *P. aeruginosa* biofilm virulence could further decrease the therapeutic success of current treatments and promote CRS disease chronicity [41]. Therapeutic strategies interfering with these cooperative processes between microbes through inhibition of nutritional exchange and signaling pathways have the potential for enhanced therapy success and better patient outcomes [42].

Furthermore, the significant increase in pyoverdine production in cocultures underscores the therapeutic potential for therapies that inhibit siderophore‐mediated iron uptake and in consequence, inhibit *P. aeruginosa* virulence in CRS. Simultaneously, the stable production of pyocyanin confirms its role as a sustained danger, and therapies designed to mitigate its oxidative activity could serve in concert with therapies designed to inhibit iron uptake [43]. Together, these therapeutic modalities could impair *P. aeruginosa's* pathogenicity and enhance CRS patient outcomes.

Protease activity also emerged as a critical variable in this investigation, with its role in mediating pathogenic *S. aureus–P. aeruginosa* interactions highlighted. Homologous cocultures displayed significantly increased protease activity, in terms of increased halo diameters in protease activity assays, indicative of enzymatic degradation of proteins [44]. The elevated activity of proteases offers a mechanistic basis for the profound disruption of epithelial barrier function documented in both TEER and permeability assays [45]. By allowing deeper tissue penetration of both bacteria and bacterial toxins, such disruption promotes inflammation and worsens disease severity in CRS. Proteases are key virulence factors responsible for the degradation of the host extracellular matrix and can indirectly modulate the immune microenvironment through the cleavage of signaling molecules and immune mediators, thereby further enhancing bacterial persistence, leading to the loss of tissue integrity and the creation of pathways for bacterial invasion [46]. Not only does enzymatic activity enable bacterial colonization, but it also aids in the evasion of immune processes, allowing continued infection and tissue damage to occur [45]. Notably, the increased activity of proteases in homologous cocultures infers that interspecies interaction in a shared host environment is specifically capable of enhancing pathogenic processes. Targeting the activity of proteases with therapy holds particularly exciting potential for CRS management.

Despite its important findings, several limitations necessitate consideration for this study. The in vitro model used, while useful for experimental controls, fails to simulate the full complexity of the in vivo human sinonasal cavity environment [47]. Moreover, given the limited availability of *S. aureus* and *P. aeruginosa* isolates obtained concurrently from the same patient, the number of biological replicates in our study was restricted. As a result, the observed phenomenon could represent an incidental finding whereby certain *S. aureus* and *P. aeruginosa* strain pairs cooperate to enhance virulence, whereas others antagonize each other's growth and pathogenic potential, and such interactions may occur independently of their coevolution within the host. Furthermore, for each patient, it remains unclear whether one of the two bacterial species was more dominant at the time of sample collection (e.g., presented with higher CFUs) or whether either species represented an active exacerbation. This uncertainty could influence the interpretation of the interaction dynamics observed in vitro. In particular, although we observed an increase in the production of pyoverdine and protease by *P. aeruginosa* in coculture, accompanied by a decrease in TEER and an increase in permeability, the relationship between these changes and barrier disruption remains correlative, and causality has not been directly verified. This study lacks an *S. aureus* monoculture control; thus, it is impossible to exclude the direct role of its secretions (such as protease, hemolysin, and other virulence factors that have been proven to damage the nasal and bronchial epithelial barrier [48]). Future research should include an *S. aureus* monoculture control and combine functional inhibition experiments (such as using protease inhibitors) to further clarify the relative contributions of different bacterial species in barrier damage. Most importantly, because of the in vitro nature of the model, the study could not evaluate potentially important host immune modulations, a critical facet of CRS pathophysiology that must not be overlooked in future studies. Future work must expand its analysis to cover additional CRS‐related pathogens and their behavior and explore, through proper in vivo studies, the role of host immunity in modulating such dynamics.

## 5 Conclusion

The present report identifies complex bacterial behavior in homologous *S. aureus* and *P. aeruginosa* cocultures, with increased epithelial barrier degradation, bacterial growth, and virulence factor expression. The coexistence of these microbes in a shared environment enhanced the pathogenicity of *P. aeruginosa*. These complex interactions speak to the therapeutic potential of personalized therapy for interfering with the cooperative microbial behavior of polymicrobial communities. In future work, one must seek out molecular underpinnings of such behavior and break microbial synergy in an attempt to seek a resolution to refractory CRS and its current and long‐standing failure in medical therapies.

## Conflicts of Interest

The authors declare no conflicts of interest.

## Supporting information




**Supporting Table 1**: TEER Differences Between *P. aeruginosa* Mono‐cultures and Co‐cultures with *S. aureus*
**Supporting Table 2**: Fluorescence intensity measurements in the basal chamber for paracellular permeability assays after 24‐h incubation with *P. aeruginosa* (PA) mono‐cultures or PA co‐cultured with *S. aureus* isolates from the same or different patients. Data represent mean fluorescence intensity (RFU) ± standard error. p < 0.05 indicates a significant increase in fluorescence intensity, while p > 0.05 indicates no statistically significant difference.
**Supporting Table 3**: Colony‐forming units (CFU) in the lower chamber of the Transwell system after 24 h of incubation. *P. aeruginosa* (PA) mono‐cultures were compared to PA co‐cultures with *S. aureus* isolates from the same patient or from different patients. Data are presented as mean ± SE. p < 0.05 indicates a statistically significant increase in bacterial proliferation compared to PA mono‐cultures while p > 0.05 indicates no statistically significant difference.
**Supporting Table 4**: Summary of *P. aeruginosa* Colony Diameters in Mono‐culture and Co‐culture Conditions with *S. aureus*
**Supporting Table 5**: Summary of *P. aeruginosa* Halo Diameters in Mono‐culture and Co‐culture Conditions with *S. aureus*
