# Supplementary material for: Patient‐Specific Coculture of S. aureus and P. aeruginosa Enhances Epithelial Barrier Disruption and Virulence in CRS
Source: Int Forum Allergy Rhinol. 2025 Sep 30;16(1):43–54. doi: 10.1002/alr.70036 (PMC12761362; doi:10.1002/alr.70036)
Supplement: Supplementary file 1 — Supporting Table 1: TEER Differences Between P. aeruginosa Mono‐cultures and Co‐cultures with S. aureus Supporting Table 2: Fluorescence intensity measurements in the basal chamber for paracellular permeability assays after 24‐h incubation with P. aeruginosa (PA) mono‐cultures or PA co‐cultured with S. aureus isolates from the same or different patients. Data represent mean fluorescence intensity (RFU) ± standard error. p < 0.05 indicates a significant increase in fluorescence intensity, while p > 0.05 indicates no statistically significant difference. Supporting Table 3: Colony‐forming units (CFU) in the lower chamber of the Transwell system after 24 h of incubation. P. aeruginosa (PA) mono‐cultures were compared to PA co‐cultures with S. aureus isolates from the same patient or from different patients. Data are presented as mean ± SE. p < 0.05 indicates a statistically significant increase in bacterial proliferation compared to PA mono‐cultures while p > 0.05 indicates no statistically significant difference. Supporting Table 4: Summary of P. aeruginosa Colony Diameters in Mono‐culture and Co‐culture Conditions with S. aureus Supporting Table 5: Summary of P. aeruginosa Halo Diameters in Mono‐culture and Co‐culture Conditions with S. aureus [file ALR-16-43-s001.docx]

**Results:**

***S. aureus* biofilm augments *P. aeruginosa* biofilm mediated mucosal barrier dysfunction when isolates are isolated from the Same Patient**

**Supplementary Table 1:** TEER Differences Between *P. aeruginosa* Mono-cultures and Co-cultures with *S. aureus*

| Patient | Earliest Significant Time Point | MD ± SE at Earliest Time Point | p-value at Earliest Time Point | MD ± SE at 24h | p-value at 24h | TEER at 24h (Mono vs. Co-culture) |
| --- | --- | --- | --- | --- | --- | --- |
| P1 | 24h | 0.325 ± 0.074 | <0.0001 | 0.325 ± 0.074 | <0.0001 | 0.742 ± 0.189 vs. 0.418 ± 0.070 (p=0.0182) |
| P2 | 6h | 0.239 ± 0.080 | 0.014 | 0.305 ± 0.080 | 0.0009 | 0.636 ± 0.094 vs. 0.331 ± 0.085 (p=0.0029) |
| P3 | 30 min | 0.199 ± 0.062 | 0.0078 | 0.314 ± 0.062 | <0.0001 | 0.608 ± 0.055 vs. 0.294 ± 0.031 (p<0.0001) |

**Supplementary Table 2:** Fluorescence intensity measurements in the basal chamber for paracellular permeability assays after 24-hour incubation with *P. aeruginosa* (PA) mono-cultures or PA co-cultured with *S. aureus* isolates from the same or different patients. Data represent mean fluorescence intensity (RFU) ± standard error. p < 0.05 indicates a significant increase in fluorescence intensity, while p > 0.05 indicates no statistically significant difference.

| Patient | Fluorescence (RFU) - PA+SA Co-culture | Fluorescence (RFU) - PA Mono | P value (Co-culture vs. Mono) | p value (Diff. Pts vs. Mono) |
| --- | --- | --- | --- | --- |
| P1 | 1236 ± 178.7 | 200.3 ± 43.09 | 0.0006 | > 0.05 |
| P2 | 1113 ± 211.5 | 197.6 ± 90.53 | 0.0028 | > 0.05 |
| P3 | 949.3 ± 226.7 | 219.0 ± 21.36 | 0.0189 | > 0.05 |

**Indirect interactions between *S. aureus* and *P. aeruginosa* biofilms Isolated from the Same Patient enhance *P. aeruginosa* Colony-Forming Units (CFU)**

**Supplementary Table 3:**Colony-forming units (CFU) in the lower chamber of the Transwell system after 24 hours of incubation. *P. aeruginosa* (PA) mono-cultures were compared to PA co-cultures with *S. aureus* isolates from the same patient or from different patients. Data are presented as mean ± SE. p < 0.05 indicates a statistically significant increase in bacterial proliferation compared to PA mono-cultures while p > 0.05 indicates no statistically significant difference.

| Patient | PA+SA (Same Pt) CFU/mL | PA Mono CFU/mL | PA+SA (Diff. Pt) CFU/mL | p-value (Same Pt vs. Mono / Diff. Pt vs. Mono) |
| --- | --- | --- | --- | --- |
| P1 | 2.69×10⁸ ± 2.50×10⁷ | 1.90×10⁸ ± 1.73×10⁷ | 1.46×10⁸ ± 3.42×10⁷ / 1.64×10⁸ ± 1.29×10⁷ | 0.0109 / > 0.05 |
| P2 | 4.89×10⁸ ± 3.47×10⁷ | 2.62×10⁸ ± 2.69×10⁷ | 2.39×10⁸ ± 2.55×10⁷ / 3.28×10⁸ ± 8.22×10⁷ | 0.0009 / > 0.05 |
| P3 | 4.28×10⁸ ± 6.74×10⁷ | 2.04×10⁸ ± 1.83×10⁷ | 2.40×10⁸ ± 3.61×10⁷ / 2.42×10⁸ ± 5.01×10⁷ | 0.0051 / > 0.05 |

**The Effect of Co-Cultured *S. aureus* and *P. aeruginosa* Isolates on *P. aeruginosa* Protease Activity in CRS Patients**

**Supplementary Table 4:** Summary of *P. aeruginosa* Colony Diameters in Mono-culture and Co-culture Conditions with *S. aureus*

| Patient | *P. aeruginosa* monoculture | Co-culture with *S. aureus* from same patient | Co-culture with *S. aureus* from different patient(s) |
| --- | --- | --- | --- |
| P1 | 1.5 ± 0.2 | 2.3 ± 0.2 (p = 0.0013) | 1.55 ± 0.3 / 1.7 ± 0.116 (p> 0.05) |
| P2 | 1.525 ± 0.096 | 2.075 ± 0.171 (p = 0.0014) | 1.425 ± 0.126 / 1.675 ± 0.096 (p> 0.05) |
| P3 | 1.575 ± 0.171 | 1.95 ± 0.1 (p = 0.0091) | 1.5 ± 0.1155 / 1.475 ± 0.25 (p> 0.05) |

# Supplementary Table 5：Summary of *P. aeruginosa* Halo Diameters in Mono-culture and Co-culture Conditions with *S. aureus*

| Patient | *P. aeruginosa* monoculture | Co-culture with *S. aureus* from same patient | Co-culture with *S. aureus* from different patient(s) |
| --- | --- | --- | --- |
| P1 | 2.0 ± 0.163 | 3.125 ± 0.189 (p = 0.0001) | 2.15 ± 0.412 / 2.2 ± 0.163 (p> 0.05) |
| P2 | 2.175 ± 0.171 | 2.85 ± 0.252 (p = 0.0044) | 2.025 ± 0.206 / 2.125 ± 0.096 (p> 0.05) |
| P3 | 2.15 ± 0.173 | 2.925 ± 0.222 (p = 0.0015) | 2.025 ± 0.171 / 2.125 ± 0.222 (p> 0.05) |
